# Pathways to the emergence of online group polarization in sports events

**DOI:** 10.1371/journal.pone.0361299

**Published:** 2026-10-27

**Authors:** Jianshan Bai, Deping Hu, Tao Wang

**Affiliations:** School of Economics and Management, Shanghai University of Sport, Shanghai, China; Government Law College, INDIA

## Abstract

Online group polarization surrounding sports events has emerged as a significant challenge for digital public opinion governance. Drawing on social combustion theory, the study identifies seven explanatory conditions: political triggers, news narrative frames, opinion leaders, capital intervention, first media, event attributes, and stakeholder participation. Using a sample of 24 recent high-profile sporting events, the study employs crisp-set Qualitative Comparative Analysis (cs QCA) to investigate the configurational pathways leading to online group polarization and to generate insights for its mitigation. The findings identify five distinct pathways through which online group polarization in sports events emerges, revealing two underlying generative mechanisms. The first is the “sensitive issues–emotional narrative” mechanism, while the second is the “non-sensitive issues–capital involvement” mechanism.

## Introduction

Group polarization, a concept originally introduced by James Stoner, refers to a group decision-making phenomenon whereby attitudes and decisions formed through group discussion become more extreme and risk-oriented than the individual positions held prior to deliberation [1]. In online environments, this phenomenon has attracted renewed scholarly attention because of the speed, scale, and interactivity of digital communication platforms [2]. Nevertheless, online group polarization is conceptually distinct from related phenomena such as cyberbullying, online violence, public opinion crises, and public criticism. Cyberbullying and online violence generally involve deliberate harmful actions directed at specific individuals, often characterized by a power imbalance [3]. Public opinion crises, by contrast, refer to the rapid emergence and widespread dissemination of negative public sentiment that threatens the legitimacy or reputation of an organization [4]. Public criticism typically denotes reasoned and issue-oriented dissent expressed without malicious intent.

However, online group polarization primarily refers to the process through which collective attitudes shift toward more extreme positions and does not require the existence of “two mutually hostile opposing factions” as a prerequisite. Specifically, two forms of online group polarization can be distinguished. First, unidirectional collective radicalization: individuals initially hold moderately critical attitudes toward the parties involved in an event, but sustained interaction within an online group gradually intensifies these attitudes into strong and extreme negative sentiments. Even in the absence of an opposing group of comparable size, this process satisfies the core definition of group polarization, namely, that the position formed through group deliberation becomes more extreme than the views individuals held before deliberation. Second, bilateral factional polarization: online participants form two identifiable and opposing blocs. Interaction within each bloc intensifies members’ existing attitudes, while hostile exchanges between the two blocs further escalate intergroup conflict, resulting in clearly differentiated opposing camps. Both forms fall within the conceptual domain of online group polarization; the primary distinction is whether polarization occurs through convergence toward a common extreme position or through the development of two competing factions of relatively comparable strength.Put simply, online group polarization can be regarded as a precursor to cyberbullying and online violence. It is often accompanied by aggressive online behaviors that, as they intensify, may gradually develop into more severe forms of cyberbullying and online violence. Therefore, examining online group polarization in the context of sporting events primarily aims to understand the evolution of such aggressive online behaviors. Accordingly, the study focuses on illustrating this evolutionary process through case analyses.

Since the early 21st century, information has increasingly become a critical factor influencing development across all sectors of society. Online platforms have created new avenues for individuals to express opinions and release emotions, while emerging technologies have fostered a new category of public opinion actors*—*netizens [5]. By leveraging the internet, these individuals have formed online communities and emerged as significant influencers of public opinion in the digital sphere.Jiang et al.(2021) argue that online community discussions in the digital sphere tend to amplify participants’ views, making them more extreme [6]. Del Vicario et al.(2016)identify group polarization as a common phenomenon in online group communication [7]. Bail et al.(2018) contend that on large-scale internet platforms, netizens can discuss shared issues and coalesce into groups. Fear of social isolation within these groups contributes to online group polarization, whereby dominant opinions intensify while weaker ones diminish [8]. Brady et al.(2023) suggest that in cyberspace, events or issues spread similarly to a virus, undergoing continuous replication, aggregation, and dissemination [9]. The rapidity, breadth, and influence of this spread increase almost geometrically, often biasing user behavior toward group polarization.Brüggemann et al.(2020) note that networked groups influence netizens’ evaluation of issues; the prevailing online atmosphere can intensify emotions during debates, leading some participants to deviate from rational judgment and adopt extreme positions [10]. Cinelli et al.(2021) argue that when collective emotions in online groups surpass rational deliberation, the group is prone to enter a blind, extreme, and irrational state, with previously undifferentiated members forming a polarized networked group [11].

In recent years, online rumors and incidents of cyberbullying associated with sporting events have become increasingly prevalent. These phenomena not only harm individuals involved but also undermine the public’s positive perception of sports and disrupt the normal functioning of the sports industry. Addressing online group polarization in the context of sporting events has thus emerged as an urgent practical challenge for researchers. However, compared to studies on general online group polarization, research specifically examining this phenomenon in sporting contexts remains limited, and investigations into “online group polarization in sporting events” are still in their early stages.Vermeer & Araujo (2019), analyzing over 4.5 million Twitter posts, found that the central role of traditional media in shaping public opinion about sporting events is gradually diminishing [5]. He also observed a negative correlation between media-based social intermediaries and public engagement, whereas individual intermediaries, such as fans and fan groups, have become the primary actors in disseminating public opinion about sports events. These actors particularly dominate discussions surrounding top-tier leagues, often collaborating to generate large-scale public opinion “storms.”Burgers et al.(2023) examined real-time Twitter commentary from professional clubs in the Dutch Eredivisie during the 2019–2020 season and reported that public opinion on sports events initially exhibits diverse attitudes due to match uncertainty [12]. As the match progresses and interactions among netizens increase during the diffusion phase, certain viewpoints attract more likes and comments, gradually forming hot topics. By the climax phase, many initially free-flowing viewpoints, influenced by the “Matthew effect” of online public opinion evolution, become polarized according to the match’s outcome.

As a precursor to cyberbullying, online group polarization in the context of sporting events has become a pressing issue that requires attention from multiple sectors of society. Against this backdrop, scholars have examined the phenomenon from various perspectives. First, some researchers argue that public discourse surrounding sporting events is closely intertwined with political issues, and that online group polarization often emerges when online communities focus on politically sensitive topics.Meier et al. (2021) contends that governments frequently use major sporting events to project a positive national image to international audiences, whereas Western non-governmental organizations often employ these events as platforms to criticize social problems in host countries [13]. Similarly, Glassel (2025) found that the hosting of the 2022 FIFA World Cup in Qatar intensified public debates in Germany concerning media practices, democratic quality, and the inclusion of minority groups [14]. Second, scholars have identified media framing as a significant driver of online group polarization during sporting events. In particular, satirical, sensationalist, or highly critical reporting styles are more likely to exacerbate polarized online discussions. Yang (2025) argues that the openness and instrumental nature of digital media enable event-related public opinion to deviate from conventional communication channels, thereby influencing its production, dissemination, and interaction processes [15]. Likewise, Brown (2023), using the Black Lives Matter movement as a case study, found that when athletes publicly endorse social movements, media coverage framed through a critical lens can provoke fan boycotts and public demonstrations [16]. Third, opinion leaders have been identified as another important factor contributing to online group polarization in sporting events. Hassan (2024) found that celebrities and other public figures exert substantial influence on discussions related to sports, cultural exchange, and sociopolitical issues, thereby shaping the direction and intensity of online debates [17]. Fourth, scholars have highlighted a range of additional factors that may contribute to online group polarization during sporting events. Beyond the commonly discussed influences of political discourse, media framing, and opinion leaders, certain characteristics of sporting events themselves may also play a role. For example, highly visible events that attract widespread public attention and emotional engagement are more likely to generate polarized opinions and intensify group-based divisions in online discussions [18].

Overall, research on online group polarization in the context of sporting events has expanded considerably in recent years, generating valuable insights and practical recommendations for the management of event-related online public opinion. Nevertheless, the existing literature exhibits several limitations. In particular, studies tend to be fragmented, focusing primarily on the effects of individual influencing factors while lacking a comprehensive examination of the mechanisms underlying online group polarization in sporting events. To address this gap and promote a more holistic understanding of the phenomenon, the study draws upon social combustion theory and previous research to identify seven key influencing factors (conditional variables) associated with online group polarization. Based on this analytical framework, 24 Chinese sports event cases were selected, and crisp-set Qualitative Comparative Analysis (cs QCA) was employed to investigate the configurational pathways and underlying mechanisms that contribute to online group polarization in sporting events.

## Materials and methods

### Research methods

Qualitative Comparative Analysis (QCA) is a methodological framework that has recently gained prominence in the social sciences, including sociology, management, and communication studies. Grounded in Boolean algebra and set theory, it seeks to identify configurations of necessary and sufficient conditions. By integrating case-oriented qualitative analysis with variable-oriented quantitative approaches, QCA facilitates the examination of complex combinations of antecedent conditions underlying specific outcomes [19]. Since its introduction by Charles C. Ragin in 1987, the method has been progressively extended from the original crisp-set QCA (cs QCA) to include fuzzy-set QCA (fs QCA), multi-value QCA (mv QCA), and temporal variants such as T QCA and TS QCA, thereby forming a comprehensive analytical framework.Among the various QCA approaches, crisp-set Qualitative Comparative Analysis (cs QCA), fuzzy-set Qualitative Comparative Analysis (fs QCA), and multi-value Qualitative Comparative Analysis (mv QCA) are the most widely used. Although each method offers distinct advantages, this study adopts cs QCA for two primary reasons. First, the study seeks to simplify the analysis of the complex social phenomenon of online group polarization in sporting events. By employing a binary classification framework based on the presence or absence of conditions, cs QCA facilitates a clear assessment of the effects of different influencing factors and reduces the risk of ambiguous interpretations. As Li Junjie (2023) argues, cs QCA not only simplifies the analysis of complex social phenomena but also preserves the essential relationships among underlying interests and related social dynamics [20]. Second, many factors associated with online group polarization in sporting events are difficult to measure objectively. For example, as noted earlier, the narrative framing adopted by news media constitutes an important influencing factor. However, its actual impact is often difficult to quantify and frequently requires researcher interpretation. In such contexts, cs QCA provides a practical methodological approach by allowing researchers to classify conditions based on theoretically informed judgments and observable social phenomena, thereby facilitating relatively robust comparative analysis.

The methodological procedure of this study comprised five stages. First, variable identification and case selection were conducted. Drawing on social combustion theory and relevant literature, seven condition variables and one outcome variable were developed based on three analytical dimensions: combustible material, oxidizer, and ignition temperature. Subsequently, 24 representative high-profile incidents associated with major sporting events between 2017 and 2024 were selected as case samples.Second, a dichotomous data matrix was constructed. Following the identification of variables, the coding criteria for each variable were systematically established, and a dichotomous dataset was developed accordingly.Third, a truth table was generated. Ragin et al. (2006) demonstrated that fuzzy-set Qualitative Comparative Analysis (fs QCA) software can effectively facilitate the construction of truth tables from dichotomous data [21]. Accordingly, the study employed fs QCA 3.0 software to generate the truth table for the crisp-set analysis.Fourth, the identified configurations were examined to address contradictory cases. A contradictory configuration was observed between Case 7 and Case 20. A re-examination of these cases revealed inaccuracies in the coding of Case 20. Therefore, the corresponding values were revised and corrected.Fifth, Boolean minimization was performed to identify the configurational pathways leading to online group polarization in sporting events.Fifth, Boolean minimization was performed to identify the configurational pathways leading to online group polarization in sporting events.Furthermore, all data collection, processing, and analysis methods used in this study comply with the terms, conditions, and data-sharing policies established by the institutions that provided the original data.

### Theoretical framework

The study is grounded in social combustion theory, an interdisciplinary framework that adapts concepts, principles, and methods from the natural sciences—particularly physics—to interpret and analyze social phenomena. Proposed by Niu Wenyuan, a leading figure in the Chinese School of Social Physics, the theory conceptualizes adverse social events through an analogy with combustion processes. It posits that three essential elements—combustible materials, oxidizers, and ignition temperature—jointly account for the emergence of such events. First, the underlying drivers of social disorder, including imbalances in human–nature relationships and conflicts in human–human interactions, are regarded as “combustible materials” that fuel instability. Second, factors such as misleading media coverage, exaggeration, rumor dissemination, malicious attacks, and the amplification of public sentiment function as “oxidizers” that intensify social unrest. Third, sudden incidents of sufficient scale and impact act as “triggers” or “ignition temperatures” that precipitate social upheaval. This framework has been widely applied in China to domains such as public crisis formation in urban agglomerations, online collective incidents, social stability early warning systems, and public opinion dynamics. Building on this theoretical foundation, the present study employs an iterative dialogue between case evidence and theory to identify causal relationships in small-N samples, thereby uncovering configurational pathways that lead to online group polarization in sporting events and clarifying its underlying mechanisms.Specifically, during the construction of the truth tables, this study identified a contradictory configurational path involving Case 7 and Case 20. Accordingly, we re-examined these cases and found that the outcome variable for Case 20 had been omitted. It was subsequently corrected.

#### Combustible material: Political triggers (PT).

According to social combustion theory, “combustible material” denotes the fundamental driving forces underlying social disorder. Within this framework, the “fuel” for risk in sporting events can be conceptualized as factors that trigger extreme social emotions among online communities, as these are the fundamental drivers of public opinion chaos. In particular, political triggers tend to draw criticism because they divert sporting events from their intrinsic purpose, thereby contributing to disorder in public discourse.For example, Olympic medal rankings are frequently interpreted as indicators of national soft power, which may provoke ideological tensions and contribute to online group polarization. A pertinent example is the controversy surrounding the frequent pre-competition drug testing of Chinese swimmers during the Paris 2024 Olympics, which generated widespread dissatisfaction among Chinese netizens and led to online group polarization. Based on this analysis, the study proposes Hypothesis 1:Political Triggers constitute a significant subset of the phenomenon of online group polarization in sports events.

Under this assumption, it is essential to establish clear coding criteria for political factors. The politicization of sporting events has long been a central topic in sports communication research and has attracted extensive scholarly attention. Drawing on the existing literature, the study identifies two criteria for determining political triggers. First, issues that extend beyond the sports domain and center on broader public political conflicts may serve as political triggers. For example, Boykoff’s analysis of media coverage of the 2022 Beijing Winter Olympics found that mainstream media outlets in Europe and the United States devoted relatively limited attention to athletic competitions, instead emphasizing geopolitical and human rights issues [22]. Consequently, public discourse was shaped primarily by political controversies rather than by athletic performance. Second, political actions undertaken by participants in sporting events may act as catalysts for public opinion. For instance, Billings and Hardin(2023) found that Moroccan players’ display of Palestinian-themed symbols during the Qatar World Cup, intended to express geopolitical positions, directly sparked public opinion conflicts between Western and Middle Eastern audiences [23].

#### Oxidizers: News narrative framework (NNF), opinion leader (OL), capital involvement (CI).

Oxidizers is a catalyst for the emergence of trending events in sports. It can stir up or provoke public sentiment, causing the popularity of a sporting event to rise steadily until it escalates into a trending event. This includes factors such as media misinformation, irrational speculation, and the pursuit of narrow interests.

The variable of news narrative framing refers to the narrative strategies employed by news disseminators when constructing and presenting news content from particular perspectives. Different narrative styles may elicit varying degrees of emotional intensity among online audiences. Bharati et al. (2026) suggests that narrative features characterized by inflammatory or satirical elements create conducive conditions for the emergence of group polarization [24]. Under the media logic that “a picture is worth a thousand words,” the expression of public opinion has become increasingly emotionalized and, at times, extreme. Driven by mechanisms such as emotional contagion and affective release, this tendency may further intensify group polarization. Moreover, within inflammatory or satirical narrative frameworks, specific details of trending events are often selectively amplified, with each new detail serving as a vehicle for the diffusion and escalation of extreme sentiments, thereby functioning as a catalyst for group polarization. Based on this reasoning, Hypothesis 2 is proposed: News narrative frameworks characterized by inflammatory or satirical language constitute a significant subset of the phenomenon of online group polarization in sports events.Under this assumption, it is essential to establish clear coding criteria for identifying inflammatory or satirical news narrative frameworks. Drawing on existing scholarship and the characteristics of news narratives, the study defines such frameworks along two dimensions. First, at the headline level, a news item is classified as employing an inflammatory or satirical narrative framework if its headline contains keywords with explicit negative emotional connotations, such as “fake” or “fraud.” For example, Pavlova (2025) found that satirical sports reporting frequently replaces objective descriptions of events with mocking or derogatory labels [25]. Accordingly, keywords that deviate from the conventions of neutral everyday language may serve as indicators of satirical narratives. Second, at the content level, a news report is considered to adopt an inflammatory or satirical narrative framework when its portrayal of an event does not accurately reflect the complete sequence of events. Specifically, reports that selectively emphasize negative aspects while omitting critical contextual or factual information fall within this category. For instance, “From Media Hype to Twitter Storm: News Explosions and Their Impact on Issues, Crises, and Public Opinion” argues that a defining feature of “media hype” is the extraction of emotionally charged fragments from events while excluding essential contextual information, thereby generating successive waves of media attention [26].

In cyberspace, emotions such as aversion, anger, and fear expressed by opinion leaders are readily transmitted to online communities, thereby triggering or amplifying these affective responses. This process contributes to the increasing salience of specific events and the diffusion of public sentiment, effectively functioning as a catalyst for online group polarization.Cha et al. (2010) define opinion leaders as individuals and organizations capable of influencing the flow of information and the focus of public attention [27]. Therefore, in this study, opinion leaders primarily refer to bloggers with a large following in cyberspace, also known as “internet celebrities.” Jin et al. (2025) indicates that the emotional framing constructed by opinion leaders in crisis contexts often contains strong affective cues and value-laden judgments, which are more easily internalized by audiences [28]. This framing subsequently shapes audience perceptions of crisis-related information, leading to varying degrees of divergence between perceived and actual conditions. Based on this reasoning, Hypothesis 3 is proposed:Opinion leaders’ expressions of negative emotions toward sporting events constitute a significant subset of online group polarization regarding such events. Under this assumption, the present study posits that the mechanism through which opinion leaders influence public discourse is analogous to that of news narrative frameworks. Specifically, the expression of negative emotions by opinion leaders can function as a form of “news,” although it lacks the structured and institutionalized processes characteristic of professional news production. Accordingly, the study considers any public statement made by opinion leaders that contains keywords with specific negative connotations to constitute an expression of negative emotion. It is important to note that these keywords need not appear in fixed positions within a statement, as this form of “news” does not follow standardized or established narrative conventions.

Sustained public attention to sporting events and objective media coverage on Weibo are important factors influencing public opinion risks [29]. From a deeper perspective, the persistence of public attention can be attributed to the logic of capital accumulation and conversion inherent in a traffic-driven digital environment. Within this framework, fan participation functions as both the primary driver and the productive force underlying capital accumulation and conversion. To maintain continuous fan engagement, commercial actors may cultivate or amplify issues that attract fan interest, even when such issues undermine the spirit of fair competition and increase the risk of group polarization. For example, a report by the China National Radio Network entitled How Does a Distorted “Fan Culture” Erode Sports, Deviate from the Spirit of Competition, and Alienate the Industry Ecosystem notes that “in pursuit of commercial interests, some brands and platforms even fan the flames.” These observations suggest that capital intervention has become an important contributing factor to online group polarization surrounding sporting events. Accordingly, the study incorporates capital intervention into the explanatory framework and conceptualizes it as a catalyst that intensifies the formation of polarized opinions. Based on this reasoning, Hypothesis 4 is proposed: capital intervention is a significant contributing factor to online group polarization in sporting events.Under this assumption, it is necessary to establish clear criteria for identifying capital intervention. Capital intervention has long been a prominent topic in research on online public opinion surrounding sporting events.Billings and Angelini (2007) found through a content analysis of Olympic television broadcasts that commercially driven media organizations often frame sporting events through nationalist narratives, frequently employing adversarial dichotomies such as “our athletes” versus “foreign opponents.” Such framing can reinforce divisions between fan groups and contribute to the polarization of online discourse [30]. Similarly, Hutchins and Rowe (2012) argue that the increasing penetration of capital into digital sports platforms encourages the prioritization of engagement-maximization strategies, including the amplification of controversial content and antagonistic interactions. These mechanisms divide fans into relatively isolated and oppositional communities, thereby accelerating the emergence of group polarization during major sporting events [31]. Based on these observations, the study conceptualizes this recurring phenomenon as a “mismatch in value claims.” Specifically, when the focus of public discussion deviates from the sporting event itself and becomes concentrated on competing value claims associated with particular athletes or fan groups, such a mismatch is considered indicative of capital intervention.It is important to emphasize that this phenomenon is also evident in fan-driven competitive behavior. To further distinguish this phenomenon from value alienation resulting from capital intervention, the study seeks to minimize the influence of organic fan dynamics by examining two lines of evidence. First, evidence of platform-driven traffic bias suggests that social media platforms have consistently promoted short videos and trending topics emphasizing rivalry between fan groups to users. Second, evidence indicates that athletes’ management teams and sponsoring brands have deliberately reinforced athletes’ public personas and connected sporting events with commercial marketing strategies.

#### Ignition temperatures: First media (FM), Event attribute (EA), stakeholder participation (SP).

Ignition temperatures refers to the catalyst that triggers major events in sports, typically denoting sudden incidents of significant scale and impact.

The term “first media” refers to the initial source through which an event is brought to public attention. In the absence of such a source, it is difficult for issues related to sporting events to generate widespread online discussion. Thus, the “first media” variable can be conceptualized as analogous to the “ignition temperature.” Generally, first media can be categorized into two primary types: unofficial reports reports and official reports. Unofficial reports refer to instances in which information about an event is first disseminated by netizens, media organizations, or other actors via social media platforms, thereby attracting public attention and stimulating discussion. In contrast, official reports refer to instances in which information is initially released by government authorities or event organizers, subsequently drawing public attention and prompting discussion.It should be noted that this study does not further differentiate among the various actors engaged in “unofficial reporting.” Empirical observations suggest that, regardless of whether such actors are ordinary users, professional media organizations, or self-media accounts, instances in which they bring public issues to attention prior to official sources are more likely to trigger online group polarization.This classification approach also enhances the feasibility of policy responses by official institutions, as it emphasizes the importance of seizing the initiative in information dissemination rather than distinguishing among different types of information sources. Cheng et al. (2017) suggests that once a trending event is disclosed and diffused into the online sphere by participants, netizens, or media organizations, it is likely to escalate into a large-scale online public opinion crisis [32]. Based on this reasoning, Hypothesis 5 is proposed: when a sporting event-related issue enters the public domain through user-generated disclosures, it is more likely to attract widespread attention and induce online group polarization.

The term “event attributes” refer to the types of issues associated with trending events. In an era dominated by attention economies, sensitive topics often attract heightened interest from self-media users and high-profile influences [33]. When a sporting event enters the public sphere, such sensitive issues are more likely to generate widespread online attention. Thus, the “event attribute” variable can be conceptualized as a “flashpoint.” Previous research by Hanna (2019), drawing on online surveys of sensitive personal topics, highlights the central role of emotion in online and qualitative research, consistent with the findings of Ian Burkitt that emotion is fundamental to social interaction [34]. Accordingly, in the context of online public opinion, “sensitive social issues” are defined as topics that are particularly likely to evoke strong emotional responses among netizens. Within sporting events, incidents involving casualties may evoke grief, violations of sportsmanship may provoke anger, and breaches of sporting ethics may generate public disapproval. All such cases fall within the category of socially sensitive issues. Based on this reasoning, Hypothesis 6 is proposed: Sensitive sports-related topics constitute a significant subset of the phenomenon of online group polarization in the sports community.Building on this framework, it is necessary to further clarify the coding criteria for sensitive issues. Specifically, four categories were identified. First, incidents involving injuries or fatalities among athletes or referees. Second, the use of performance-enhancing drugs by athletes. Third, instances of referees engaging in biased officiating. Fourth, instances of nationalistic behavior by athletes or referees.

The term “stakeholder participation” refers to the involvement of the parties directly involved in or related to an event. In the context of high-profile sports events, the responses of those involved often spark a temporary sensation among online communities and become headline news eagerly covered by various online media outlets. Consequently, the “subject participation” variable is likened to a “flashpoint.” Therefore, the study proposes Hypothesis 7: Participation by key stakeholders is a significant subset of the phenomenon of online group polarization in sports events.If individuals directly involved in a major sports event (such as family members or friends) participate in discussions about the topic, this is considered active participation.

### Outcome variable: Extreme social emotions (ESS)

Extreme social emotions refer to high-intensity emotional states that emerge collectively in response to specific social issues and deviate substantially from the normative distribution of public emotions. Liu (2020) argues that anger, fear, and disgust are the most prevalent forms of extreme social emotions, which may manifest in diverse ways [35]. On the one hand, such emotions can be expressed directly through abusive or inflammatory language, making them relatively easy to identify. On the other hand, they may be embedded within the deeper structure of a text through sarcasm, implicit evaluations, conditional expressions, or seemingly objective factual statements. Consequently, extreme social emotions are difficult to measure using objective criteria alone and often require researchers to exercise interpretive judgment during the coding process.

To address this issue, the study moved beyond a single quantitative threshold based on the proportion of oppositional comments and refined the ESS coding rules in two respects. First, we compared a random sample of early-stage comments from periods of relatively low traffic with a random sample of highly visible comments posted during the peak of a trending topic. If the early-stage comments predominantly expressed moderate and rational criticism, whereas comments during the peak period contained widespread explicit abusive language, implicit sarcasm, derogatory remarks, or other forms of extreme sentiment, the case was considered to meet the core criterion for group polarization and was coded as 1; otherwise, it was coded as 0. Second, we assessed whether identifiable opposing opinion groups emerged during the peak period. If two relatively balanced and mutually hostile opinion camps could be clearly distinguished in the comments and both sides contained comments accusing or attacking the other, the case was coded as 1; otherwise, it was coded as 0.

## Case selection and assignment

### Case selection

Benoît Rihoux and Charles C. Larkin argue that case selection in Qualitative Comparative Analysis (QCA) should adhere to the principles of similarity and diversity; that is, while ensuring case homogeneity, researchers should also strive to maintain heterogeneity. Accordingly, the present study adopts the following criteria for case selection [36].

First, case comparability was ensured. In the case selection process for QCA, the initial step is to define the scope of investigation, thereby establishing the boundaries for case inclusion. Accordingly, this study focused on high-profile sports event incidents that received coverage from national- or central-level media outlets. Rihoux and Ragin argue that similarity among cases should be carefully considered during case selection. High-profile sports event incidents are inherently characterized by substantial social influence. By restricting the sample to incidents covered by national or central-level media, the study ensured that all selected cases generated significant social impact and attracted widespread public attention. Compared with alternative selection criteria, this approach more effectively guarantees the comparability of cases.

Second, case diversity was maintained. Rihoux and Ragin further emphasize that diversity constitutes another key criterion in case selection.Therefore, while maintaining case homogeneity, the cases selected for this study encompass a diverse range of sports events, including track and field, soccer, and skiing competitions, conducted between 2017 and 2024 and varying in scale from mega-sport events to major and local sporting events.

Third, the case selection followed the principles of MDSO and MSDO research designs. Specifically, the sample included both MDSO configurations, in which similar combinations of antecedent conditions produce different outcomes, and MSDO configurations, in which identical outcomes arise from different combinations of antecedent conditions. By incorporating both types of configurations, the study enhanced the explanatory power and robustness of the QCA analysis.

Based on these three criteria, a total of 24 representative high-profile sports event incidents occurring between 2017 and 2024 were selected as case samples for analysis, as presented in Table 1.

**Table 1:** Sports events hot events case table.

ID	Event Name	Event Scale	Project
1	Sudden Death Incident at the First Yinchuan International Marathon	Local Sport Events	Long-distance running
2	The Incident Involving Runners Stranded at the Xuedou Mountain 100-Mile Trail Race in Jiangnan	Local Sport Events	Trail running
3	The Su Yiming Scoring Controversy at the Beijing Winter Olympics	Mega Sport Events	Skiing
4	The August 8 Tianmenshan Cycling Race Rider Plunge Incident in China	Local Sport Events	Bicycle
5	The Tragedy in Baiyin, Gansu, China: 21 People Killed	Local Sport Events	Trail running
6	Sudden Death Incident at the 2024 Asian Youth Badminton Championships	Major Sport Events	Badminton
7	The Beijing Half Marathon Scandal	Local Sport Events	Long-distance running
8	The “Crossing the Finish Line” Incident at the Hong Kong-Zhuhai-Macao Marathon	Local Sport Events	Long-distance running
9	Incident of chaos at the CBA Liaoning-Guangdong Finals	Major Sport Events	Basketball
10	The Chinese National Team’s 0–7 Defeat in the 23rd FIFA World Cup Asian Qualifiers	Mega Sport Events	Soccer
11	The 2023 Chengdu Universiade Opening Ceremony Sparks Hot Debate	Major Sport Events	General
12	The Women’s Singles Table Tennis Final at the Paris Olympics Sparks Hot Debate	Mega Sport Events	Table tennis
13	The Paris Olympics Gymnastics Team’s Mistake Sparks Hot Debate	Mega Sport Events	Gymnastics
14	Women’s 10m Platform Final at the 2023 Chinese Diving Championships	Major Sport Events	Diving
15	Sudden Death Incident at the Lingshan 100 International Mountain Trail Challenge	Local Sport Events	Trail running
16	Sudden Death Incident at the Shenqiu Marathon in Henan, China	Local Sport Events	Long-distance running
17	The “Hammer Throw Incident” Involving a Qatari Athlete at the Hangzhou Asian Games	Major Sport Events	Hammer throw
18	The Incident of Messi Missing the Friendly Match in Hong Kong	Local Sport Events	Soccer
19	Wu Yanni Fails to Reach Semifinals at Paris Olympics, Sparking Online Debate	Mega Sport Events	Hurdles
20	Post-match altercation involving the Dalian Yingbo Guangzhou team	Local Sport Events	Soccer
21	The Incident Where Wang Chuqin’s Racket Was Stepped On at the Paris Olympics	Mega Sport Events	Table tennis
22	The Controversy Over Transgender Boxers Competing in the Paris Olympics	Mega Sport Events	Boxing
23	The 2018 Chinese Marathon Flag-Handing Incident	Local Sport Events	Long-distance running
24	The 2019 Xiamen Marathon Impersonation Incident	Local Sport Events	Long-distance running

### Case assignment

The coding process in this study comprised three stages. First, coding rules for all variables were established based on the preceding analysis (see Table 2). Second, to ensure coding reliability, three researchers—including the author—independently assigned codes after reaching agreement on the coding scheme. Any discrepancies were subsequently resolved through discussion until consensus was achieved.At the same time, the study used SPSS 27.0 to calculate the consistency prior to reaching consensus. The Fleiss’ kappa coefficients ranged from 0.822 to 1.00, all exceeding 0.75 (the coefficients for PT, NNF, OL, CI, FM, EA, SP, and ESS were 0.862, 0.862, 1.00, 0.822, 0.944, 1.00, 1.00, and 0.825, respectively), indicating a high level of consistency and that the coding rules and scoring were reasonably applied. Third, the final coding results for all variables are presented in Table 3. Among these cases, NNF and OL were both equal to 1 in several instances. To assess the independence of NNF and OL, the study used SPSS 27.0 to calculate the phi coefficient for the two variables. The phi coefficient is commonly interpreted alongside the chi-square test. However, when more than 20% of the cells in a chi-square test have expected frequencies below 5, the Pearson chi-square approximation may be unreliable; in such cases, statistical significance should be assessed using Fisher’s exact test. The results showed that the phi coefficient between NNF and OL was 0.408, with a chi-square statistic of approximately 4.0 and a Fisher’s exact test p-value of 0.129. Notably, 50% of the cells had expected frequencies below 5. Therefore, although the phi coefficient indicated an association between NNF and OL, the Fisher’s exact test did not provide statistically significant evidence of an association. Accordingly, the analysis does not support a statistically significant relationship between NNF and OL, and the two variables can be treated as independent for the purposes of this analysis.

**Table 2:** Variable assignment rules and instructions.

Variable type	Combustion conditions	Variable name	Set of targets	Explanation of the Determination	Code assignment
Conditional variable	Combustible material	PT	The Alienation of the Issue	Public discourse has focused on political conflicts	1
			Political behavior	Athletes or referees expressing political views	1
			Non-political factors	Other factors contributing to online public opinion	0
	Oxidizers	NNF	The Distortion of News Headlines	Keywords in the title that convey specific negative connotations (such as “counterfeit” or “fake”)	1
			The Distortion of News Content	The content presents only specific negative material and omits key factual information	1
			The news narrative is normal	No discrepancy was observed between the news headlines and their content	0
		OL	Expressing Negative Emotions	The views publicly expressed by opinion leaders carry a specific negative emotional connotation	1
			None	No opinion leaders have expressed views carrying specific negative connotations	0
		CI	Platform Traffic Allocation	Social media platforms used to continuously push short videos and trending topics featuring factional conflicts to users	1
			Brand Marketing Integration	Athletes’ management teams and brand sponsors deliberately craft and promote the athletes’ public personas, and tie sporting events to brand marketing	1
			None	There were no signs of significant capital intervention	0
	Ignition temperatures	FM	Unofficial Announcement	The incident first drew public attention and sparked heated discussion following unofficial reports	1
		EA	Official Announcement	The incident first drew public attention and sparked heated discussion following official reports	0
			Sensitive issues	First, accidents resulting in injury or death involving athletes or referees. Second, athletes using performance-enhancing drugs. Third, instances of referees engaging in biased officiating. Fourth, instances of nationalistic behavior by athletes or referees.	1
			Non-sensitive topics	No clear trend of radicalization in public opinion has been observed	0
		SP	Allegations by the parties involved	Those directly involved in the incident or related parties have voiced their opinions, pointing out problems with the event	1
			The party did not allege the incident	The individuals involved did not point out any problems with the event	0
Outcome variable	ESS		Unidirectional collective radicalization	Extreme Shifts in the Attitudes of Online Groups Over Time	1
			Bilateral factional antagonistic polarization	Divisions Within Online Communities	1
			No extreme social sentiment has emerged	No clear trend toward radicalization in public opinion has emerged	0

**Table 3:** Variable assignment list.

ID	ESS	PT	NNF	OL	CI	FM	EA	SP
1	0	0	1	0	0	0	1	0
2	0	0	1	0	0	1	0	1
3	1	1	1	1	1	0	1	0
4	0	0	0	1	0	0	1	1
5	1	0	1	1	0	1	1	1
6	1	0	1	1	0	0	1	1
7	1	0	1	1	0	1	1	0
8	1	1	1	1	0	1	1	0
9	1	0	1	1	1	0	0	0
10	1	0	1	1	0	0	1	1
11	0	1	1	1	0	0	0	0
12	1	0	1	0	1	0	0	0
13	1	0	1	1	1	0	0	1
14	1	0	1	0	1	0	0	1
15	0	0	0	0	0	1	1	1
16	0	0	0	0	0	1	1	1
17	0	0	0	0	0	1	1	0
18	1	1	1	1	1	1	0	1
19	0	0	0	0	1	0	0	1
20	1	0	1	1	0	1	1	0
21	1	0	1	1	1	0	0	1
22	1	1	1	1	0	0	1	1
23	1	1	1	1	0	1	1	1
24	1	0	0	1	0	1	1	1

## Results

### Single-condition necessity test

The study primarily uses the fsQCA 3.0 software to conduct single-condition necessity tests and configuration path analysis. The single-condition necessity test examines the consistency and coverage of a single antecedent condition with respect to the outcome.Consistency refers to the proportion of cases in which a given condition (or combination of conditions) is associated with the same outcome. When this index is greater than 0.90 and 0.80, it is generally considered a necessary and sufficient condition for the outcome variable to occur.Coverage indicates the extent to which a condition (or combination of conditions) accounts for the outcome: values closer to 1 indicate greater explanatory power.The results of the single-condition necessity test are presented in Table 4 (Round the result to four decimal places).

**Table 4:** Single variable necessity test.

Conditional variable	ESS		~ ESS	
	Consistency	coverage rate	Consistency	coverage rate
PT	0.3125	0.8333	0.8750	0.3889
NNF	0.9375	0.8333	0.6250	0.8333
OL	0.8750	0.8750	0.7500	0.7500
CI	0.4375	0.8750	0.8750	0.4375
FM	0.5000	0.6667	0.5000	0.3333
EA	0.6250	0.6667	0.3750	0.3333
SP	0.5625	0.6429	0.3750	0.3000

As shown in Table 4, substantial differences exist in the consistency and coverage scores of the conditional variables. First, regarding the occurrence of ESS, the consistency values of NNF and OL exceed 0.90 and 0.80, respectively, suggesting that they may be regarded as necessary and sufficient conditions under conventional QCA criteria. However, this study identified an exception in Case 24, where ESS occurred despite the absence of divergence between news headlines and content, indicating that NNF was not present. This anomaly can be attributed to the fact that the case emerged amid widespread skepticism expressed by opinion leaders, such as “Dingding Runner,” and other members of the running community. As a result, the event reached a sufficiently high level of “ignition temperature,” with FM, EA, and SP all present. This finding suggests that NNF should be considered a near-necessary rather than a strictly necessary condition for online group polarization in sports events. Second, with respect to cases in which ESS did not occur, the consistency values of PT and CI exceeded 0.80, indicating that these conditions do not constitute significant subsets of online group polarization in sports events. On this basis, the study proceeds with subsequent counterfactual analysis.

### Pathways

After completing the single-condition necessity test, the study followed the recommendations of Rihoux and Ragin by setting the consistency threshold at 0.80—above the commonly recommended level of 0.75—and the case frequency threshold at 1, in order to identify configurations of conditional factors [36]. This procedure yielded complex, intermediate, and parsimonious solutions for combinations of conditions.Building on this, and guided by Fiss’s “core–periphery model of typologies,” the study adopts intermediate solutions as the primary basis for interpretation and simple (parsimonious) solutions as a supplementary reference [37]. Conditions that appear in both intermediate and simple solutions are defined as core conditions, whereas those appearing only in intermediate solutions are classified as peripheral conditions.Furthermore, following the notation proposed by Lakin and Fiss, a solid black circle (•) indicates the presence of a condition, a crossed hollow circle (⊗) denotes the absence of a core condition, and a blank space indicates that a condition may be either present or absent (see Table 5). At the same time, to assess the robustness of the data analysis, the study conducted an additional robustness test by increasing the consistency threshold from 0.80 to 0.90 while holding all other conditions constant. The results showed that the findings remained unchanged under the two threshold specifications, suggesting that the results of this study are robust to variations in the consistency threshold.

**Table 5:** Pathways to the emergence of online group polarization in sports events.

Variable	Path 1	Path 2	Path 3	Path 4	Path 5
PT		⊗			⊗
NNF	•	•	•	•	
OL	•		•	•	•
CI	⊗	•			⊗
FM		⊗	⊗	•	•
EA	•	⊗	•	⊗	•
SP			⊗	•	•
Case	5,6,7,8,10,20,22,23	9,12,13,14,21	3	18	5,24
Raw coverage	0.5	0.3125	0.0625	0.0625	0.125
Unique coverage	0.375	0.3125	0.0625	0.0625	0.0625
Overall coverage	1				
Overall consistency	1				

Note: • indicates a condition that must be present in the combination path (• denotes a core condition, • denotes a boundary condition); ⊗ indicates a condition that need not be present in the combination path (⊗ denotes a core condition, ⊗ denotes a boundary condition), denoted by the symbol ~).

#### Path 1: NNF×OL×~CI×EA.

As shown in Table 5, Path 1 achieved a raw coverage of 0.50 and encompassed the largest number of cases (Cases 5, 6, 7, 8, 10, 20, 22, and 23), making it the most prevalent pathway leading to online group polarization in sports events. In this configuration, NNF, OL, and EA function as core present conditions, whereas CI serves as a core absent condition. This pathway can be interpreted as follows: in sports events involving sensitive issues, even in the absence of capital intervention, inflammatory or satirical news narratives, combined with negative sentiment amplified by influential opinion leaders and celebrities, can rapidly trigger intense public reactions. Under such circumstances, the expression of negative opinions by relevant actors is sufficient to ignite widespread emotional responses, ultimately resulting in online group polarization.

Second, Case 6 is used to illustrate the specific mechanism underlying this pathway. On June 30, 2024, Zhang Zhijie, a young Chinese badminton player, collapsed and subsequently died during the 2024 Asian Junior Badminton Championships. Because the incident involved the death of a young national athlete, it attracted widespread public attention immediately following official media coverage. Although the initial posts sparking the incident were mostly moderate appeals, as news media ran satirical reports and opinion leaders got involved, sensational headlines containing phrases such as “sudden death,” “tragedy,” “the family’s despair,” and “17 years old” further heightened public engagement. As a result, subsequent comments on the incident were, without exception, rife with sarcastic condemnation of the event organizers and sports federations. These comments concealed widespread, deeply negative sentiment, which was expressed through rhetorical questions and implicit accusations, thereby fueling the emergence of online group polarization.

On July 1, a prominent social media influencer, “News Sister” (25.717 million followers on Douyin), posted a video alleging that neither cardiopulmonary resuscitation (CPR) nor an automated external defibrillator (AED) had been administered during the initial two-minute emergency response period. These allegations triggered widespread criticism among netizens and contributed to the subsequent development of online polarization surrounding the incident.Subsequently, on July 3, 2024, Zhang Zhijie’s sister publicly expressed dissatisfaction with the event organizers on Weibo and stated her intention to pursue accountability. This statement rapidly amplified public emotions, prompting widespread criticism of the Indonesian Badminton Association. Some netizens also directed their dissatisfaction toward the Chinese Badminton Association, thereby creating a critical trigger for the escalation of online group polarization.

Throughout the process, numerous online influencers and public figures continued to express dissatisfaction with what they perceived as delayed and inadequate medical treatment during the event, sustaining public attention and negative sentiment. For example, influencers such as “Jiaic” (306,000 followers on Weibo) and “Global Lens” (733,000 followers on Weibo) reiterated on August 17 that Zhang Zhijie’s death resulted from delayed medical intervention. Such continued discussions helped maintain the salience of the incident and prolonged its influence within online public discourse.It is clear that if authorities mishandle a sporting event involving a sensitive issue, even without the involvement of capital interests, the combination of inflammatory news narratives and the views of opinion leaders can instantly ignite public sentiment—and have a lasting impact—the moment those directly affected voice their dissatisfaction.

Based on the dual-mode ESS coding criteria, Case 6 represents a form of attitude radicalization within a single online community. A random sample of comments from both the early low-traffic period and the peak of the trending topic supports this classification. During the initial stage, participants formed a relatively unified online community centered on sympathy for the deceased athlete, with no clearly differentiated opposing fan groups or competing value-based camps. As information was repeatedly circulated, inflammatory news narratives intensified emotional responses, and opinion leaders repeatedly expressed negative sentiments, the group’s overall attitude gradually shifted toward extreme resentment, sarcasm, and accusatory judgments directed at the event organizers and sports governing bodies. Although no opposing faction with a comparable volume of discourse emerged, the collective shift toward increasingly extreme attitudes following sustained interaction within the online group demonstrates a clear instance of group polarization.

#### Path 2: ~PT×NNF×CI×~FM×~EA.

As shown in Table 5, Path 2 exhibits a raw coverage of 0.3125 and encompasses the second-largest number of cases (Cases 9, 12, 13, 14, and 21), making it another prevalent pathway leading to online group polarization in sports events. In this configuration, NNF and CI function as core present conditions, whereas FM and EA serve as core absent conditions, and PT acts as a peripheral absent condition.This pathway can be interpreted as follows: once a sporting event enters the public sphere through official channels, its scale, visibility, or established status within the sporting community may attract substantial public attention. Under the influence of sensationalized or satirical news narratives, together with intervention by capital interests, the event is likely to evoke intense collective emotions and facilitate online group polarization. Notably, this outcome may occur even when the event does not involve sensitive social issues or political conflicts, and regardless of whether opinion leaders or directly involved actors express negative sentiments.

Second, Case 12 is employed to illustrate the specific mechanism underlying this pathway. On August 3, 2024, the women’s singles table tennis final was held at the Paris Olympic Games. Following Chen Meng’s victory, extensive controversy emerged across online communities, exhibiting a pronounced pattern of online group polarization. An examination of the event’s evolution suggests that audience reactions at the venue provided the initial conditions for subsequent online disputes, functioning as the “fuel” for polarization.

From the perspective of the event itself, competition for a championship represents the essence of elite sports and does not inherently constitute a sensitive social issue. Moreover, because both finalists represented China, the event was not characterized by political conflict. Under such circumstances, media coverage would be expected to adhere to principles of objective reporting. However, during the dissemination process, certain reports displayed elements of headline–content divergence or sensationalized framing, which contributed to the escalation of public controversy.Simultaneously, a series of event-related topics, including “#ChenMengWins,” “#ChenMengJadeNecklace,” and “#LiuGuoliangFrowns,” rapidly gained traction on social media platforms. The proliferation of these topics created opportunities for the expansion and interaction of fan communities, facilitating the transformation of loosely connected supporters into more organized fan groups. As discussions intensified, online discourse increasingly deviated from the sporting event itself and became centered on competing identity claims and value judgments.

Consequently, a substantial volume of unverified and misleading information concerning Chen Meng and Sun Yingsha circulated online. Some users engaged in personal attacks against athletes and disseminated content that generated broader social controversy, thereby amplifying online conflict and exerting negative effects on both the athletes and the Chinese table tennis team. For example, a company executive surnamed Tang repeatedly alleged that Chen Meng’s victory resulted from favoritism within the sports system, while a netizen surnamed He fabricated claims regarding the athlete’s relationship with her coach. These allegations intensified public disputes and further fueled online polarization. Although the dissemination of such content declined following legal intervention by public security authorities, traces of the extreme sentiments generated during the controversy continued to influence online discussions for an extended period.It is evident that, fueled by both the “news narrative framework” and “capital intervention,” even events that are not sensitive topics or matters deliberately concealed by the authorities—that is, those with a low “ignition threshold”—can still trigger online group polarization.

Based on the dual-mode ESS coding criteria, Case 12 represents a typical instance of bilateral factional polarization. Driven by capital intervention and algorithmic traffic allocation, the previously unified table tennis fan community rapidly fragmented into two mutually antagonistic groups: supporters of Sun Yingsha and supporters of Chen Meng. A stratified sample of comments posted during the peak period of the trending topic showed that the two camps generated relatively comparable volumes of discourse and repeatedly directed hostile remarks, sarcastic comments, and personal attacks toward the opposing athlete and her supporters.

#### Path 3: NNF×OL×~FM×EA×~SP.

As shown in Table 5, Path 3 has an original coverage of 0.0625 and encompasses Case 3, representing a relatively rare generative pathway leading to online group polarization in sports events. In this configuration, NNF, OL, and EA function as core present conditions, FM serves as a core absent condition, and SP acts as a peripheral absent condition. This pathway suggests that inflammatory and satirical news narratives, combined with the emotional amplification effects of opinion leaders, can foster online group polarization even when a sporting event is introduced to the public through official channels. When the event involves a sensitive issue, these factors are likely to trigger extreme collective emotions among online audiences. Moreover, in such circumstances, positive public statements made by the parties involved are often insufficient to mitigate or reverse the polarization process surrounding the sporting event.

Second, a review of the sequence of events in Case 3 helps clarify the underlying logic of this pathway. The scoring controversy involving Su Yiming during the Beijing Winter Olympics on February 7, 2022, triggered widespread criticism among netizens, largely because both the gold medalist, Max Parrot, and the judging panel were from Canada. Although Su Yiming was the only athlete to successfully complete a 1800-degree jump (five consecutive 360-degree rotations) and Parrot committed a grab-related error during his run, Su’s final score remained lower than Parrot’s. Because the event was broadcast live, online media outlets, opinion leaders, and netizens began discussing the controversy before the competition had even concluded. Amplified by sensationalized and satirical news narratives, as well as emotional mobilization by opinion leaders, the incident rapidly rose to the top of online trending lists, attracting increasing public attention and participation. As a result, a large-scale wave of online criticism quickly emerged.

On the morning of February 10, Iztok Šumatić, the chief referee for the event, gave an interview in which he acknowledged that a scoring error had occurred while suggesting that the judges may have had legitimate reasons for their decisions. Rather than easing public concerns, this statement appeared to validate the suspicions of many netizens and further intensified online polarization surrounding the event. Although Su Yiming’s coach publicly urged the public to stop criticizing the judges later that evening, the appeal failed to alleviate the increasingly extreme sentiments expressed online. Throughout the controversy, derivative hashtags such as “#SuYimingScoredIncorrectly,” “#SuYimingLikesChiefReferee’sMistake,” “#SuYimingDeservesIt,” “#SuYiming’sMagnanimity,” and “#TsangChiWaiSpeaksOut” continued to proliferate across social media platforms. Instead of subsiding, the controversy escalated further, with some netizens extending their criticism to Su Yiming’s coach and even to the nation itself.

Based on the dual-mode ESS coding criteria, Case 3 represents polarization through the radicalization of attitudes within a single online community. Initially, participants were united around the central issue of perceived unfair officiating against Chinese athletes. During the early stage, no two clearly differentiated opposing factions had emerged based on fan loyalty or national identity; instead, participants constituted a relatively unified online community characterized by mild disappointment and uncertainty. As inflammatory media narratives circulated and multiple sports opinion leaders repeatedly steered the discussion toward negative sentiment, the group’s hostility toward the Canadian refereeing team and foreign athletes progressively intensified. The limited number of neutral voices defending the referees did not develop into an opposing faction with comparable discursive weight, partly because of social conformity pressures within the group and differential algorithmic exposure. Overall, the case demonstrates a unidirectional shift from relatively moderate attitudes toward increasingly extreme negative sentiment within a single online community.

#### Path 4: NNF×OL×FM×~EA×SP.

As shown in Table 5, Path 4 has an original coverage of 0.0625 and encompasses Case 18, representing another relatively rare generative pathway leading to online group polarization in sports events. In this configuration, NNF, OL, and FM function as core present conditions, SP serves as a peripheral present condition, and EA acts as a core absent condition. This pathway suggests that when problems arise during a sporting event and event organizers fail to respond promptly, subsequent dissemination of the incident by netizens through social media platforms can trigger online group polarization. Even when the incident does not involve a sensitive issue, sensationalized and satirical news narratives, combined with the emotional amplification effects of opinion leaders, can foster polarized online discussions surrounding the sporting event.

Second, a review of the sequence of events in Case 18 helps clarify the underlying logic of this pathway. The controversy surrounding Lionel Messi’s absence from the Hong Kong friendly match on February 4, 2024, emerged after Inter Miami had stipulated in its pre-match contractual agreement that key players, including Messi, Jordi Alba, and Luis Suárez, would participate for at least 45 minutes unless prevented by injury. In addition, promotional materials for the event led spectators to expect Messi’s appearance. However, neither Messi nor Suárez took the field during the match. Faced with a substantial discrepancy between expectations and reality, spectators at the venue demanded refunds and shared their dissatisfaction through posts and images on social media platforms. The incident was subsequently reported extensively by major online media outlets, generating widespread public attention and criticism.

Despite post-match explanations provided by Inter Miami head coach Gerardo Martino and event organizer Tatler Asia, online polarization surrounding the incident persisted. Although Tatler Asia ultimately offered a half of ticket refund, the controversy continued to generate long-term repercussions. Evidence of these lingering effects includes the removal of Messi-related content from the official website of the Chinese Football Association and the suspension of cooperation between the Chinese Football Association and the Argentine Football Association.

An examination of the incident reveals three key factors contributing to the emergence of online group polarization. First, inflammatory and satirical news narratives disseminated by numerous online media outlets, combined with the emotional amplification effects of opinion leaders, rapidly increased the visibility of the controversy. Even in the absence of definitive video evidence or official confirmation, a predominantly one-sided climate of public criticism had already emerged, providing the primary impetus for online polarization. Second, the event organizers failed to respond within a critical communication window. Their first press conference was not held until the evening of February 5, and they did not adequately address the financial losses incurred by spectators. Consequently, they were unable to effectively reduce the conditions conducive to the escalation of group polarization. Third, the incident involved multiple stakeholders, including the Hong Kong SAR Government, which represented the interests of local spectators, Inter Miami, and Tatler Asia. Each party adopted a distinct position, and no consensus was reached. For example, on February 6, 2024, Hong Kong SAR Chief Executive John Lee stated that he was deeply disappointed that Messi had not appeared as expected and that the event organizers had failed to satisfy the government’s requirements. Such public statements further intensified public attention and contributed to the continued escalation of online polarization.

Based on the two forms of online group polarization defined by the ESS coding rules, Case 18 represents a typical instance of attitude radicalization within a single online community. Participants initially focused on the event organizer’s alleged breach of contract and misleading marketing practices, forming a relatively unified opinion community around shared demands for compensation and official accountability. Early online comments primarily expressed reasonable requests for refunds and mild skepticism regarding the organizer’s marketing claims. As sensationalist media narratives continued to amplify public attention and negative sentiment was reinforced by opinion leaders from multiple sectors, including government officials, sports bloggers, and entertainment influencers, the online community gradually shifted from relatively moderate skepticism to increasingly extreme accusations, calls for boycotts, and disparaging remarks directed at Messi, the soccer club, and the event organizers.

#### Path 5: ~PT×OL×~CI×FM×EA×SP.

As shown in Table 5, Path 5 has an original coverage of 0.125 and encompasses Cases 5 and 24, representing a relatively uncommon pathway leading to online group polarization in sports events. In this configuration, OL, FM, and EA function as core present conditions; SP serves as a peripheral present condition; and CI and PT operate as a core absent condition and a peripheral absent condition, respectively. This pathway suggests that even in the absence of capital intervention and political contestation, online group polarization may emerge when a sporting event involves a sensitive issue and event organizers or hosts fail to respond promptly during a critical communication window. Under such circumstances, netizens may identify behaviors perceived as inconsistent with the principles of sportsmanship or ethical conduct. Amplified by the influence of opinion leaders and the active participation of actors directly involved in the incident, collective online emotions can intensify and gradually evolve into online group polarization.

Second, Case 24 is used to illustrate the specific development of this trajectory. The case concerns firefighter Xu Yihao, who participated in the 2019 Xiamen Marathon on January 6, 2019, by running under another participant’s registration. Initially, the incident attracted little public attention. However, following the race, Xu shared his marathon experience on social media. Although his intention was to promote positive values, netizens soon discovered that the bib number he used (J29739) did not match his registered identity. Meanwhile, the event organizers failed to detect and address the violation in a timely manner. As skepticism intensified among opinion leaders, including “Dingding Runner,” and other members of the running community, the incident rapidly gained public attention and evolved into a highly contentious online discussion, ultimately triggering group polarization surrounding the event.It was not until January 14, 2019, that the Chinese Athletics Association issued the “Notice on Additional Penalties for Hu Xin, Liu Haiqing, Xu Yihao, Chu Yingjun, and Wen Shengyong in the 2019 Jianfa Xiamen Marathon.” The notice imposed a two-year suspension on Xu Yihao from all events sanctioned by the Chinese Athletics Association, in addition to a lifetime ban from participation in the Xiamen Marathon. Following the implementation of these formal sanctions, online public sentiment gradually subsided.

The incident involved neither ideological conflict nor capital influence, and it was not amplified by sensationalist reporting in online media. Instead, it was driven primarily by the discourse of opinion leaders. This finding suggests that when the “ignition temperature” reaches a sufficiently high level, online group polarization in sporting-event contexts can emerge even in the absence of ideological confrontation, capital intervention, or media sensationalism.

Based on the dual-mode ESS coding criteria, Case 24 represents a typical instance of bilateral factional polarization. After the disclosure of the alleged use of a proxy runner to participate under another runner’s bib number, the previously unified running community divided into two opposing camps with relatively comparable influence. One camp comprised professional runners, race organizers, and sports ethics bloggers, who argued that bib transfers and proxy running, regardless of the participant’s subjective intent, violated the principle of fair competition and directed criticism toward Xu Yihao’s conduct. The other camp comprised ordinary spectators and supporters of the firefighter, who emphasized his original intention to raise public awareness and criticized the “lifetime ban” as excessively harsh and lacking compassion.

## Discussion

Overall, although the configurations differ in their specific combinations of conditions, both NNF and OL consistently emerge as core conditions across nearly all pathways. NNF is absent only in Path 5, while OL is absent only in Path 2. This pattern indicates that inflammatory or satirical news narratives (NNF) and the negative emotional expressions of opinion leaders (OL) function as near-necessary catalysts for online group polarization in sporting events. In their absence, the emergence of large-scale extreme social sentiment within online sports communities is unlikely.

At the same time, CI as a critical oxidizer in this process. On the one hand, the underlying logic of capital intervention is that platforms, brands, MCNs, and other commercial actors, motivated by the pursuit of traffic monetization, intensify event-related hype through sustained promotional practices, including profit-oriented manipulation of public opinion and the pursuit of narrow commercial interests, thereby serving as important intermediary forces. On the other hand, in the absence of ignition sources—such as conflicts among athletes or unexpected incidents—as initial triggers for public discussion, capital intervention lacks the necessary content resources for amplification and faces difficulties in generating large-scale polarizing topics. Therefore, capital intervention does not possess the capacity to independently initiate public opinion conflicts; rather, it primarily functions to amplify existing controversies and intensify disorder once an initial trigger has emerged. Accordingly, capital intervention is conceptualized as a fuel rather than an ignition source.

Interestingly, EA and CI emerge as critical factors in explaining online group polarization during sporting events. Pathways 1, 2, and 5 represent the most prevalent configurational mechanisms associated with this phenomenon. Across these pathways, EA and CI consistently function either as core present conditions or core absent conditions. Moreover, their effects appear to be functionally equivalent, as the two conditions never occur simultaneously within the same configuration. This pattern suggests that online group polarization in sporting events follows two distinct triggering logics.The first is the “sensitive issue–emotional narrative” logic. Under this logic, when a sporting event involves a socially sensitive issue, online users are more likely to develop emotional identification with the event. In such circumstances, inflammatory news narratives and the negative emotional expressions of opinion leaders can rapidly resonate with audiences, thereby facilitating group polarization.The second is the “non-sensitive issue–capital intervention” logic. This logic suggests that when a sporting event lacks inherent social sensitivity, attention should be directed toward whether public attention has been distorted by external influences. Under the influence of capital intervention, misaligned value claims may intensify competition among fan communities, giving rise to “fan-circle” conflicts that ultimately trigger group polarization.

Several limitations of this study should be acknowledged. First, although the binary coding approach employed in cs QCA enhances analytical clarity and facilitates configurational comparisons, it may oversimplify complex phenomena and result in the loss of nuanced information. Future research could adopt multi-value QCA (mv QCA) or fuzzy-set QCA (fs QCA) to capture greater variation in conditional variables and provide a more refined analysis. Second, the study primarily examines online group polarization in sports events from a macro-level perspective, focusing on the mechanisms underlying its emergence. As a result, micro-level factors have received comparatively limited scholarly attention. Future research should therefore examine these factors in greater depth to develop a more nuanced understanding of their roles and mechanisms. Third, the study focuses primarily on online phenomena in China. While the Chinese context is significant given the country’s large population and extensive online user base, the extent to which the findings can be generalized across different cultural and institutional contexts remains uncertain. Future studies should therefore examine the proposed relationships in diverse national settings to assess the robustness and cross-cultural applicability of the findings. Fourth, the study develops its analytical framework solely based on the social combustion theory. Although this approach ensures consistency in identifying antecedent conditions, it may overlook the complementary insights provided by other theoretical perspectives, particularly the differentiated explanations offered by competing theories. Therefore, future research should systematically explore the diverse mechanisms underlying group polarization in sports event networks by integrating insights from multiple theoretical perspectives. Finally, the publicly available online comment data used in this study may be incomplete because platform algorithms or government regulation may have resulted in the removal of dissenting opinions. This limitation may be particularly evident in high-profile sporting events such as Case 12, where the observed polarization, reflected in the radicalization of attitudes within a single online community and the absence of opposing camps, may potentially result from content regulation rather than accurately reflect the underlying distribution of public opinion. Accordingly, the findings associated with Path 2 should be interpreted with caution. To assess the potential impact of data incompleteness on QCA pathway findings, we conducted a robustness test by increasing the consistency threshold from 0.80 to 0.90. The results remained consistent across the two analyses, suggesting that the QCA pathway findings demonstrate a certain degree of robustness. Future research should further examine how data regulation may shape online polarization surrounding sporting events, thereby providing additional insights into the governance of a healthy online ecosystem for sports-related discussions.

## Conclusion and suggestion

Drawing on Social Combustion Theory and employing the cs QCA method, this study investigated the multiple configurational pathways leading to online group polarization during sporting events. Through a configurational analysis of seven conditional variables (PT, NNF, OL, CI, FM, EA, and SP), five equifinal pathways leading to extreme social sentiment (ESS) were identified. Building on these findings, further analysis revealed that online group polarization in sporting events generally operates through two distinct triggering mechanisms, which can be differentiated according to the sensitivity of the event-related topic.

First, in addressing sensitive issues, strict oversight should be exercised over inflammatory and satirical news narratives, as well as the dissemination of negative emotional expressions by opinion leaders. In contemporary media environments, emotional appeals often exert a stronger influence on public opinion than the presentation of objective facts. When emotions overshadow truth and prejudice supersedes objectivity, news stories are more likely to be disseminated competitively and sensationally, thereby fostering extreme social sentiments within online communities. Consequently, when sensitive issues emerge during sporting events, event organizers should proactively take the lead in information dissemination. By providing timely, objective, and comprehensive explanations of the causes of such issues, assuming appropriate responsibility, and implementing effective remedial measures, organizers can reduce the risk of misinformation and narrative distortion, gain the support of opinion leaders, and ultimately mitigate online polarization within sports event communities.

Second, with respect to response mechanisms for non-sensitive issues, greater efforts should be directed toward addressing disorderly behavior within sports fan communities. For example, during the 2022 UEFA Women’s EURO, UEFA launched the “Respect Initiative,” collaborating with social media platforms such as Twitter and TikTok to employ automated detection systems and human moderation to monitor and remove harmful content, including abusive and discriminatory comments directed at players, coaches, and officials, in real time. During the tournament, approximately 60% of non-compliant posts were removed by the platforms, while 119 cases of harmful content were directly addressed during the final stage of the competition. These measures demonstrate the importance of proactive governance in maintaining a healthy online environment. Accordingly, mitigating fan-community disorder surrounding sporting events requires the optimization of content moderation technologies, a reassessment of the notion of algorithmic neutrality, and the incorporation of value-oriented indicators, such as sportsmanship and online civility, into recommendation algorithms. Such measures can increase the visibility of positive content and contribute to the development of a governance framework characterized by effective human–machine collaboration.

## References

[1] Stoner JAF. A comparison of individual and group decisions involving risk. Cambridge, MA: Massachusetts Institute of Technology. 1961. http://hdl.handle.net/1721.1/11330

[2] Sunstein CR. Republic: Divided Democracy in the Age of Social Media. Princeton, NJ: Princeton University Press. 2017.

[3] Kowalski RM, Giumetti GW, Schroeder AN, Lattanner MR. Bullying in the digital age: a critical review and meta-analysis of cyberbullying research among youth. Psychol Bull. 2014;140(4):1073–137. doi: 10.1037/a0035618 24512111

[4] Zhang L, Dong P, Zhang L, Mu B, Yang A. A systematic literature review of crisis management in online public opinion: evolutionary path and implications for China. Kybernetes. 2024;54(9):4866–901. doi: 10.1108/k-10-2023-1985

[5] Vermeer SAM, Araujo TB. Keep the Ball Rolling: Information Diffusion within Large Sports-Related Networks through Social Mediators. Communication & Sport. 2019;8(6):803–24. doi: 10.1177/2167479519841868

[6] Jiang S, Wang H. Group Polarization Based on Agent Emotional Characteristics and Credibility. Complexity. 2021;2021:9983541. doi: 10.1155/2021/9983541

[7] Del Vicario M, Vivaldo G, Bessi A, Zollo F, Scala A, Caldarelli G. Echo chambers: emotional contagion and group polarization on Facebook. Scientific Reports. 2016;6:37825. doi: 10.1038/srep37825 PMC5131349 27905402

[8] Bail CA, Argyle LP, Brown TW, Bumpus JP, Chen H, Hunzaker MBF, et al. Exposure to opposing views on social media can increase political polarization. Proc Natl Acad Sci U S A. 2018;115(37):9216–21. doi: 10.1073/pnas.1804840115 30154168 PMC6140520

[9] Brady WJ, McLoughlin KL, Torres MP, Luo KF, Gendron M, Crockett MJ. Overperception of moral outrage in online social networks inflates beliefs about intergroup hostility. Nat Hum Behav. 2023;7(6):917–27. doi: 10.1038/s41562-023-01582-0 37037990

[10] Brüggemann M, Elgesem D, Bienzeisler N, Gertz HD, Walter S. Mutual group polarization in the blogosphere: Tracking the hoax discourse on climate change. International Journal of Communication. 2020;14:24–43.

[11] Cinelli M, De Francisci Morales G, Galeazzi A, Quattrociocchi W, Starnini M. The echo chamber effect on social media. Proc Natl Acad Sci U S A. 2021;118(9):e2023301118. doi: 10.1073/pnas.2023301118 33622786 PMC7936330

[12] Burgers C, Beukeboom CJ, Smith PA, van Biemen T. How live Twitter commentaries by professional sports clubs can reveal intergroup dynamics. Computers in Human Behavior. 2023;139:107528. doi: 10.1016/j.chb.2022.107528

[13] Meier HE, Mutz M, Glathe J, Jetzke M, Hölzen M. Politicization of a Contested Mega Event: The 2018 FIFA World Cup on Twitter. Communication & Sport. 2019;9(5):785–810. doi: 10.1177/2167479519892579

[14] Gläßel C, Scharpf A, Edwards P. Does sportswashing work? First insights from the 2022 World Cup in Qatar. The Journal of Politics. 2025;87(1):388–92. doi: 10.1086/730728

[15] Yang Y, Zhang Y, Shi B. Representation, Attribution, and Purification: A Study on the Dissemination of Competitive Sports Public Opinion in the Digital Media Environment. Frontiers in Sociology. 2025;10:1591882. doi: 10.3389/fsoc.2025.1591882 PMC12427031 40949883

[16] Brown DK, Mourão RR, Ganguli T. Black Lives Matter on the Ground and in Sports: Varied Influences of Delegitimizing News Coverage on Self-Perceived Knowledge and Support for Protests. International Journal of Communication. 2025;19:19.

[17] Hassan AA, Wang J. The Qatar World Cup and Twitter sentiment: Unraveling the interplay of soft power, public opinion, and media scrutiny. International Review for the Sociology of Sport. 2024;59(5):679–704. doi: 10.1177/10126902231218700

[18] Huang H, Ruan W-Q, Zhang S-N. The Evolutionary Characteristics and Process Mechanisms of Online Public Opinion for Major Sports Event Emergencies. Sage Open. 2024;14(1). doi: 10.1177/21582440241237054

[19] Di Paola N, Chari S, Iannacci F, Kraus S. Configurational theory in business and management research: Status quo and guidelines for the application of qualitative comparative analysis (QCA). Technological Forecasting and Social Change. 2025;211:123907. doi: 10.1016/j.techfore.2024.123907

[20] Junjie L, Ke W, Bei Z, Xin D, Meng Q, Bangfan L. Influence analysis and promotion countermeasures of green finance policy in China-Traceability based on grounded theory and validation using the csQCA method. PLoS One. 2023;18(5):e0285862. doi: 10.1371/journal.pone.0285862 37200300 PMC10194979

[21] Ragin CC, Drass KA, Davey S. Fuzzy-Set/Qualitative Comparative Analysis 2.0. Tucson, AZ: Department of Sociology, University of Arizona. 2006.

[22] Boykoff J. Framing the Games: US Media Coverage of the Beijing 2022 Winter Olympics. Communication & Sport. 2022;12(1):19–39. doi: 10.1177/21674795221122938

[23] Billings AC, Hardin M. Maximized Sporting Events for Maximized Communicative Impact: The Role of Megasporting Events in 2023. Communication & Sport. 2023;11(6):1039–41. doi: 10.1177/21674795231204072

[24] Bharati R, Bharti J, Dehalwar V. A novel fuzzy logic-based hybrid framework for detecting fake news. Discov Computing. 2026;29(1). doi: 10.1007/s10791-025-09882-x

[25] Pavlova KA. Illocutionary Indicators of Sarcasm in the Genre of Sports Analytical Articles. Russian Social and Humanitarian Studies. 2025;17(2):158–75. doi: 10.12731/2077-1770-2025-17-2-508

[26] Vasterman P. From media hype to twitter storm: News explosions and their impact on issues, crises and public opinion. Taylor & Francis. 2025.

[27] Cha M, Haddadi H, Benevenuto F, Gummadi K. Measuring User Influence in Twitter: The Million Follower Fallacy. ICWSM. 2010;4(1):10–7. doi: 10.1609/icwsm.v4i1.14033

[28] Jin B, Zou M, Wei Z, Guo W. How to Find Opinion Leader on the Online Social Network?. Applied Intelligence. 2025;55(7):624. doi: 10.1007/s10489-025-06525-y

[29] Deng Y, Wang H, Wu Y. Public Opinion and Chinese Exports: Evidence from Twitter Sentiment Analysis. Journal of the Asia Pacific Economy. 2026;31(1):1–30. doi: 10.1080/13547860.2024.2403512

[30] Billings AC, Angelini JR. Packaging the Games for Viewer Consumption: Gender, Ethnicity, and Nationality in NBC’s Coverage of the 2004 Summer Olympics. Communication Quarterly. 2007;55(1):95–111. doi: 10.1080/01463370600998731

[31] Hutchins B, Rowe D. Sport beyond television: The internet, digital media and the rise of networked media sport. New York: Routledge. 2012.

[32] Cheng Y, Huang Y-HC, Chan CM. Public relations, media coverage, and public opinion in contemporary China: Testing agenda building theory in a social mediated crisis. Telematics and Informatics. 2017;34(3):765–73. doi: 10.1016/j.tele.2016.05.012

[33] Wang G, Wang Y, Liu K, Li J. Multidimensional influencing factors of public opinion information dissemination in social media: Evidence from Weibo dataset. Int J Mod Phys B. 2019;33(31):1950375. doi: 10.1142/s0217979219503752

[34] Hanna E. The emotional labour of researching sensitive topics online: considerations and implications. Qualitative Research. 2018;19(5):524–39. doi: 10.1177/1468794118781735

[35] Liu B. Sentiment Analysis: Mining Opinions, Sentiments, and Emotions. Cambridge: Cambridge University Press. 2020.

[36] Rihoux B, Ragin CC. Configurational Comparative Methods: Qualitative Comparative Analysis (QCA) and Related Techniques. Thousand Oaks, CA: SAGE Publications. 2009.

[37] Fiss PC. Building Better Causal Theories: A Fuzzy Set Approach to Typologies in Organization Research. AMJ. 2011;54(2):393–420. doi: 10.5465/amj.2011.60263120

